# Effect of Intranasal Corticosteroids on Intraocular Pressure and Nerve Fiber Layer Thickness: A Cross-Sectional Study at a Tertiary Care Hospital in Western Saudi Arabia

**DOI:** 10.7759/cureus.13146

**Published:** 2021-02-05

**Authors:** Hani Z Marzouki, Rahaf K AlThomali, Lujain Hefni, Nawaf Almarzouki, Faris Alhejaili, Mazin Merdad, Faisal Zawawi, Talal Alkhatib

**Affiliations:** 1 Otolaryngology–Head and Neck Surgery, King Abdulaziz University, Jeddah, SAU; 2 Ophthalmology, King Abdulaziz University, Jeddah, SAU; 3 Medicine, King Abdulaziz University, Jeddah, SAU

**Keywords:** rhinitis, intranasal corticosteroid, intraocular pressure, glaucoma

## Abstract

Background

Rhinitis represents a global health problem, affecting 10%-20% of the population in Saudi Arabia. Topical intranasal corticosteroids (INCS) are widely used by otolaryngologists to treat patients with rhinitis for long periods. Although the effects of orally administered corticosteroids on intraocular pressure (IOP) and lens opacity are well established, the impact of INCS is not well defined. In the present study, we aimed to assess the effect of using INCS on IOP over a six-month period in patients with rhinitis.

Methodology

In this study, a questionnaire was distributed to 93 patients diagnosed with rhinitis in the Ear, Nose, and Throat Clinic of King Abdulaziz University Hospital, Saudi Arabia, between February and July 2019. Thereafter, each patient was evaluated in the Ophthalmology Clinic with optical coherence tomography of the optic nerve using Cirrus HD-OCT (Carl Zeiss Meditec, Inc., Dublin, CA, USA) optic disc scans, visual acuity testing, and Goldmann applanation tonometry. Pearson correlation coefficients and two-tailed tests of significance were used to assess the relationships between variables.

Results

All 93 patients were using a dose of two puffs at least twice daily for each nostril. Their IOPs, as depicted by Goldman applanation tonometry, were found to be within normal limits. Retinal nerve fiber layer thickness was also found to be normal in 95% of the participants.

Conclusions

Our study showed no correlation between INCS and IOP. As an increase in IOP can lead to glaucoma, our data demonstrate the safety profile of INCS use. For patients with rhinitis, this finding could change compliance to medication and reduce the burden of the disease.

## Introduction

Rhinitis is an inflammatory disorder of the nasal mucosa. It has been hypothesized that after the process is triggered, it leads to an inflammatory cascade mediated via immunoglobulin E [1]. The main symptoms of rhinitis are rhinorrhea, sneezing, nasal itching, and nasal congestion, with congestion being one of the most bothersome symptoms because it is strongly associated with poor quality of life and has a significant negative impact on productivity [2]. It is not uncommon for conditions such as nasal polyps, asthma, and atopic dermatitis to co-exist with rhinitis. Pharmacotherapy and immunotherapy are the initial preferred treatment choices for these inflammatory conditions. Because of their anti-inflammatory properties, the drugs of choice for first-line treatment of rhinitis are intranasal corticosteroids (INCS) [3].

In 2014, the Ministry of Health in Saudi Arabia established recommendations for the effective use of INCS in treating perennial and seasonal rhinitis [4]. They are also prescribed to reduce airway edema, decrease mucus production, restore nasal breathing, and reduce recurrence of upper airway inflammation [5]. Thus, INCS are widely used in upper airway inflammatory conditions.

Moreover, several types of INCS are available for the treatment of rhinitis. Classic INCS include beclomethasone dipropionate, budesonide, flunisolide, fluticasone propionate, mometasone furoate, and triamcinolone acetonide. All are efficacious in treating seasonal rhinitis and as a prophylaxis for perennial rhinitis. The basic mechanism by which these drugs work is by relieving nasal congestion and reducing itching, rhinorrhea, and sneezing, the most common symptoms in the early and late phases of allergic responses [6].

The pharmacological action of INCS can target multiple pathways and provide effective anti-inflammatory effects. This can lead to inhibition of inflammatory cells and chemical mediators such as leukotrienes and prostaglandins, which are involved in the entire allergic process. In addition, corticosteroids act by increasing the synthesis of lipocortin-1, which inhibits phospholipase A2 and prevents the production of lipid mediators of inflammation. Mediators that are indirectly inhibited by administration of corticosteroids include histamine, kinins, platelet-activating factor, and substance P [7].

Safety of intranasal steroids

Intranasal steroids are widely used for acute and chronic inflammation of the upper airway, and therefore the safety profile of these drugs needs to be thoroughly elaborated. The main safety issues with the use of steroids include suppression of growth in the short and long term, an increase in ocular pressure, and suppression of the hypothalamic-pituitary-adrenal axis. Because patients with asthma use both inhaled corticosteroids (ICS) and systemic steroids, these drugs may have additive adverse effects [6]. However, in the case of intranasal steroids, the benefits outweigh the risks. Still, they should be used judiciously, taking into consideration the patient’s physiological and medical data. Because pharmacodynamics vary from individual to individual, the establishment of a thorough safety profile is critical [8].

There is no clinical evidence that intranasal steroids suppress the hypothalamic-pituitary-adrenal axis. Clinical studies on growth have shown that, except for beclomethasone dipropionate, none of the second-generation intranasal steroids have an adverse impact on growth [9]. Pharmacokinetically, first-generation ICS have significant first-pass hepatic metabolism and higher oral bioavailability, thus explaining the difference between the effects of the two generations [10]. However, larger studies are needed to develop a more detailed safety profile of intranasal steroids.

Ocular safety

Because intranasal steroids are commonly used for rhinitis, ocular safety has recently become an important research question. In the clinical trials published within the last three years, it is clear that lowering of intraocular pressure (IOP) in cases of severe ocular hypertension reduces the chances of developing glaucoma. This means that regulation of ocular pressure is necessary to maintain normal pressure within the eyes and to delay the progression of glaucoma. Thus, intranasal steroids should be administered cautiously in cases where the patient already has ocular hypertension [11].

The impact of the increase in IOP on the eyes is sudden and causes mechanical stress and immediate ischemic effects on the retinal nerve fiber layer (RNFL). Chronic elevation of IOP has been suggestive of primary open-angle glaucoma. It may also lead to chronic vision loss [12].

Moreover, prolonged use of intranasal steroids has been reported to cause posterior subcapsular cataract. Various studies have investigated the normal levels of IOP and eye function. IOP has been noted to have a circadian rhythm related to plasma cortisol levels and fluctuations in intraocular levels. An alteration in one parameter can occur if there are fluctuations in the other. This circadian rhythm can alter with age, medications, and systemic and local factors. According to literature, IOP may increase when steroids are administered through systemic, nasal, or topical routes, and therefore, the extent to which these corticosteroids influence IOP remains controversial [13]. The aim of this study was to evaluate the relationship between INCS use and IOP to assess patient safety and potentially improve compliance to reduce the disease burden.

## Materials and methods

Study design and participants

We performed a cross-sectional study from February to July 2019 that included 93 patients. All participants were diagnosed with rhinitis and were 15 years of age or older. Treatment with INCS for at least the previous six weeks was the inclusion criterion. All participants were recruited from the Outpatient Department of King Abdulaziz University Hospital after obtaining their verbal informed consent. Excluded from the study were patients who were previously diagnosed with intraocular hypertension (n = 9), ocular trauma/surgery, glaucoma, or other eye diseases, as well as those who were currently using systemic steroids.

Study method

A validated questionnaire was developed and administered in the Ear, Nose, and Throat Clinic. The questionnaire dealt with three types of data: demographics; the use of INCS, including the number of puffs used, the number of times used per day, and the number of weeks that the participants used them; and the Rhinitis Control Assessment Test.

After the completion of the questionnaire, each patient was assessed with optical coherence tomography (OCT) of the optic nerve using Cirrus HD-OCT (Carl Zeiss Meditec, Inc., Dublin, CA, USA) optic disc scans. Cirrus HD-OCT with the optic disc cube 200 × 200 protocol was implemented for all patients, along with visual acuity testing and and Goldmann applanation tonometry. The Goldmann tonometer was calibrated on the basis of the guidelines provided by Steven et al. [14] Mean IOP measurements were used in the analysis. The number of puffs of INCS used along with the frequency of usage was also determined.

Statistical analysis

Race, gender, and smoking status were analyzed using descriptive statistics, including frequencies and percentages. The mean, median, mode, standard deviation, variance, and range for all other variables were calculated. Pearson correlations and two-tailed tests of significance were performed for Goldmann applanation tonometry and OCT and groups of patients by number of puffs of INCS used, number of times per day of use, and number of weeks of use. Statistical analysis depicted the significance of differences within and between groups.

## Results

This study included 93 rhinitis patients, 52.7% (n = 49) females and 46.2% (n = 43) males. Overall, 67% of the participants were Saudi nationals. Among the participants, 78% were non-smokers, 15% were smokers, and 7% were ex-smokers. Thus, most participants were females and non-smokers. The mean age of the participants was 35 years. The responses recorded were analyzed carefully for completeness and relevance.

The severity of rhinitis among the participants was assessed using the Rhinitis Control Assessment Test. A score lower than 21 suggests that rhinitis symptoms are not well controlled. In this study, the symptoms of 87 (94%) patients were not controlled.

**Figure 1:**
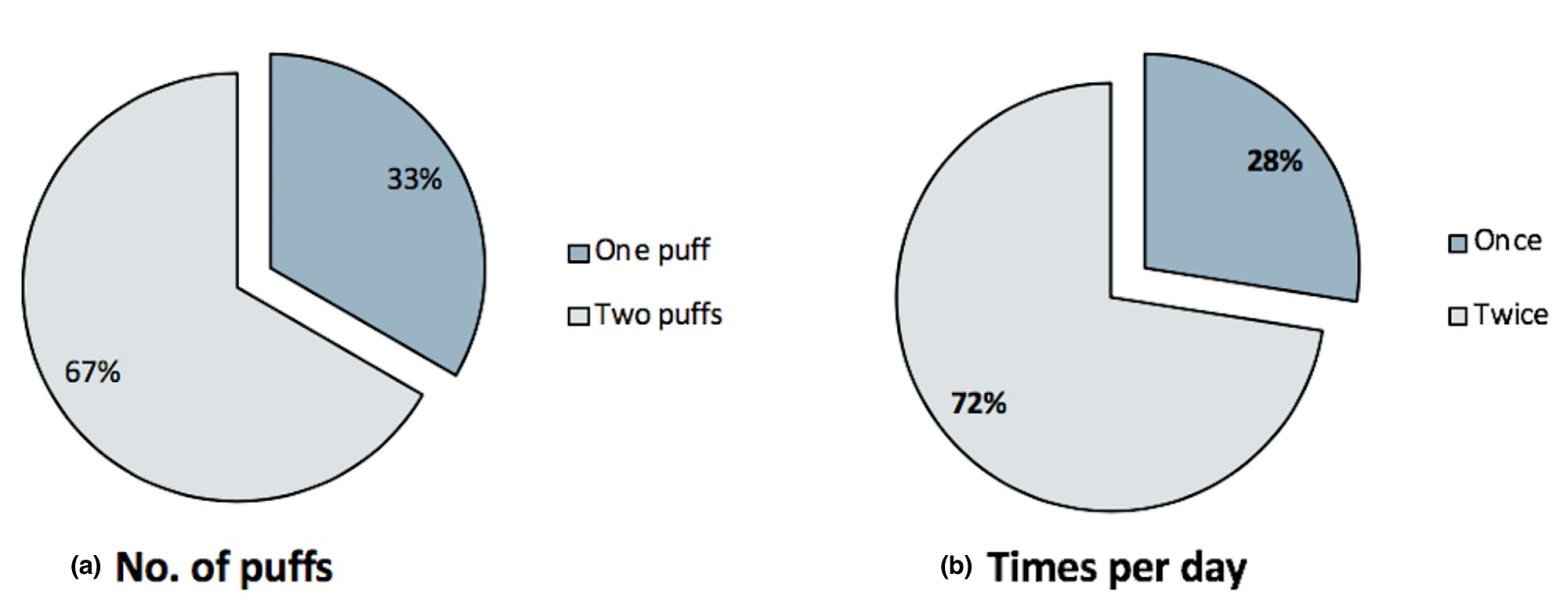
Percentage of patients using one or two INCS puffs in each nostril (a) and number of times per day (b). INCS, intranasal corticosteroid

Patient compliance with treatment was appraised by the number of puffs administered to each nostril and the number of times per day. Notably, most of our patients were using two puffs in each nostril (67%) twice daily (72%), as shown in Figure 1.

**Figure 2:**
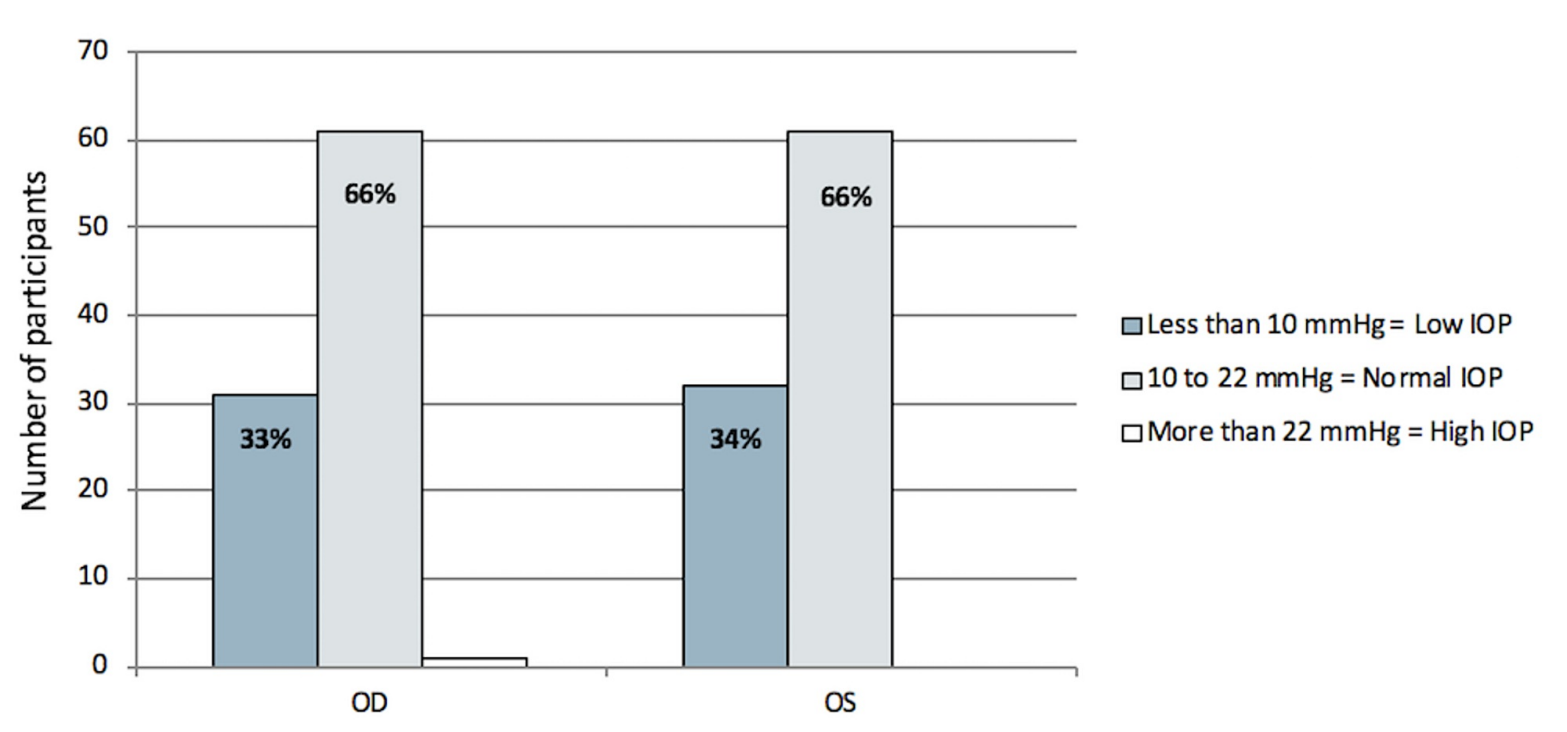
IOP results from Goldmann applanation tonometry of the OD and OS of the patients. IOP, intraocular pressure; OD, right eye; OS, left eye

Goldmann applanation tonometry was performed for both eyes to determine IOP. Figure 2 shows that 66% of the patients were within the normal range for both eyes.

**Figure 3:**
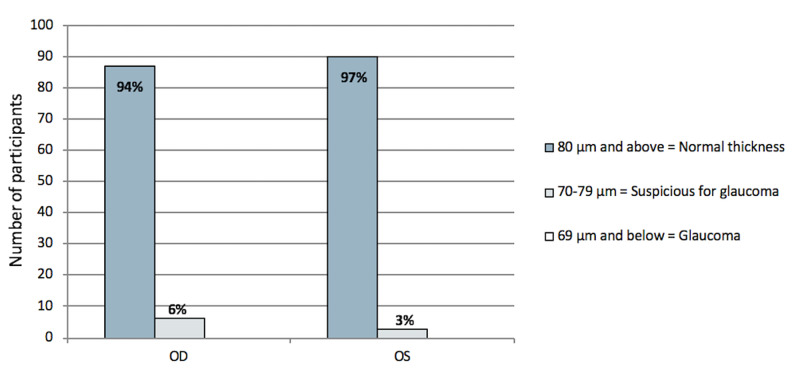
Optical coherence tomography results of the OD and OD of the patients. OCT, optical coherence tomography; OD, right eye; OS, left eye

OCT was used to measure RNFL thickness. Among the samples, 90% of the results were in the normal range, whereas 6% were suspicious for glaucoma. However, none of the cases were categorized as being in the glaucomatous range. OCT results are shown in Figure 3.

Pearson coefficient correlations showed that there were no statistically significant correlations between tonometry results and steroid usage or between the average RNFL thickness and steroid usage (Table 1).

**Table 1:** Pearson coefficients and P-values for Goldmann applanation tonometry and OCT results in relation to INCS usage. OCT, optical coherence tomography; INCS, intranasal corticosteroid

	INCS usage		
	Number of puffs	Times per day	Number of weeks
Goldmann applanation tonometry			
Right eye			
Pearson correlation	-0.039	0.057	0.050
P-value	0.711	0.587	0.634
Left eye			
Pearson correlation	-0.045	-0.155	0.232
P-value	0.668	0.138	0.025
OCT			
Right eye			
Pearson correlation	-0.074	-0.049	-0.003
P-value	0.484	0.641	0.977
Left eye			
Pearson correlation	0.094	0.114	-0.016
P-value	0.370	0.277	0.879

## Discussion

To our knowledge, the literature lacks studies concerning the effect of intranasal steroids on IOP in patients with rhinitis in Saudi Arabia. We concluded from our cohort of patients that the probability of an increase in ocular pressure secondary to the administration of INCS was very low for the test period. Optical nerve tomography, which was used to examine the thickness of the RNFL of both the right and left eyes, showed that for 95% of the participants, the results were within the normal zone, that is, 80 mm or more. The results of only 63% of the participants placed them in the suspicious category of between 70 and 79 mm. None of the participants were in the glaucomatous range, in which the thickness of the retinal nerve is 69 mm or less.

IOP varies from individual to individual. In our study, we examined the IOP of 93 participants (186 eyes). The normal ocular pressure ranges between 12 and 22 mmHg, irrespective of the central corneal thickness correction, which can affect the readings obtained from different contact and non-contact tonometers. In ideal circumstances, ocular pressure greater than 22 mmHg is considered to be a risk factor for the development of glaucoma in the long term [15]. The mean value of the Goldmann applanation tonometry results was 13.50 ± 3.359 mmHg for the right eye and 13.08 ± 3.112 mmHg for the left eye. These results fit well within the normal range for IOP.

Furthermore, the questionnaire revealed that most patients used an average of two puffs twice a day for each nostril for an average of 100 weeks. The standard deviation and variance in the usage of puffs and the number of times per day was acceptable, but the values for the usage of puffs for 100 weeks was not statistically acceptable because of a high standard deviation of 213.03.

The Pearson coefficient for Goldmann applanation tonometry suggested that there was no significant correlation between the tonometry test and the use of steroids. Likewise, the use of ICS was not a risk factor for structural changes in the optic nerve associated with glaucoma. No correlations were found between RNFL thickness and the use of steroids, which suggests that there are other factors responsible for reduction in the thickness of the RNFL and increasing IOP, thereby resulting in glaucoma. In addition, we found no correlation between the duration of ICS use and changes in RNFL thickness over time. The high P-value suggests that the results were not significant at a confidence interval of 95%. Dereci et al. [16] conducted a study that demonstrated no change in peripapillary RNFL thickness in children with asthma who were treated with an inhaled steroid. No study in the literature has suggested a relation between ICS and RNFL thickness in patients with rhinitis.

Ahmadi et al. [5] conducted a systematic review to study the impact of the administration of intranasal steroids on IOP and lens opacity. The results showed that there was no increase in IOP or development of glaucoma in 4376 patients included in 10 randomized control trials. All of the clinical trials focused on the impact of various new and old intranasal steroids for various durations from 2 to 104 weeks, and described the differences between test and control participants. The safety profile of intranasal steroids in this systematic review suggests that these steroids are low-risk alternatives for managing upper tract respiratory inflammation. IOP can change based on the duration of the administration of intranasal steroids and the physiological condition of the patients. The current recommendations suggest that a two-week review period is required before commencing non-parenteral corticosteroid use [3].

A similar systematic review and meta-analysis conducted by Valenzuela et al. [17] revealed that there was no statistically significant elevated risk of increased IOP in participants who used INCS when compared with those who received a placebo. The incidence of glaucoma was absent in 2,837 subjects with a confidence interval of 95%. Even the development of a posterior subcapsular cataract was not found in patients who were administered steroids compared with those who received a placebo. Overall, the studies included in the meta-analysis suggested that the use of INCS does not lead to an increase in IOP [17].

Yenigun et al. [18] developed a pilot study to understand the impact of topical nasal steroids administered in patients with rhinitis and dry eyes. This study was conducted for six weeks and demonstrated that administration of nasal steroids for rhinitis significantly improved symptoms of dry eye without affecting IOP. However, the results were not significant in the Schirmer I and tear breakup time tests. This study revealed that oral and eyedrop administration of corticosteroids may be more potent and harmful than nasal administration. These results, therefore, support the safety of intranasal steroids [18].

On the other hand, the long-term impact of intranasal steroids on IOP was studied by Mohd Zain et al. [19]. The results showed that the mean IOP was slightly higher in the intranasal test group (15.24 ± 2.31 mmHg) than in the control group (13.91 ± 1.86 mm Hg). This finding suggests that prolonged use of intranasal steroids can have an impact on the IOP in patients who are treated with these drugs for rhinitis.

Our study suggests that there is no direct relation between the use of intranasal steroids and IOP elevation. This may be because intranasal steroids are formulated for local delivery and therefore their systemic absorption is restricted. Newer steroids such as fluticasone propionate, mometasone furoate, ciclesonide, and fluticasone furoate are no less than ideal INCS because of their excellent pharmacodynamics and pharmacokinetic parameters. The affinity for the glucocorticoid receptor is high, providing higher potency and specificity for these drugs and reducing adverse effects through avoidance of first-pass metabolism. These INCS also provide a sustained-release drug effect, which reduces the required daily dosage of the drugs [3].

Nonetheless, the current study has some limitations. First, it is a cross-sectional study with a relatively small sample of patients. Therefore, a future study with a larger number of patients is warranted to fully support our findings. Second, we relied on direct questioning with no objective documentation to assess compliance with medical therapy.

## Conclusions

This cross-sectional study demonstrated that the safety profile of intranasal steroids is defined with considerable statistical significance. Similar studies have outlined the safety profile of intranasal steroid use, thereby providing assurance that there is no relationship between intranasal steroid use and an increase in the IOP. Larger cohort studies are required to evaluate the long-term association between INCS and IOP and RNFL thickness.

## References

[1] Pawankar R, Mori S, Ozu C, Kimura S (2011). Overview on the pathomechanisms of allergic rhinitis. Asia Pac Allergy.

[2] Abdulrahman H, Hadi U, Tarraf H (2012). Nasal allergies in the Middle Eastern population: results from the "Allergies in Middle East Survey". Am J Rhinol Allergy.

[3] Varshney J, Varshney H (2015). Allergic rhinitis: an overview. Indian J Otolaryngol Head Neck Surg.

[4] Al Rayes H, Al Enazi F, Al Rayes H (2014). Allergic rhinitis. Ministry of Health of Saudi Arabia and McMaster University Clinical Practice Guidelines on the allergic rhinitis. Saudi Arabia.

[5] Ahmadi N, Snidvongs K, Kalish L, Sacks R, Tumuluri K, Wilcsek G, Harvey R (2015). Intranasal corticosteroids do not affect intraocular pressure or lens opacity: a systematic review of controlled trials. Rhinology.

[6] Trangsrud AJ, Whitaker AL, Small RE (2002). Intranasal corticosteroids for allergic rhinitis. Pharmacotherapy.

[7] LaForce C (1999). Use of nasal steroids in managing allergic rhinitis. J Allergy Clin Immunol.

[8] Sheth K (2008). Evaluating the safety of intranasal steroids in the treatment of allergic rhinitis. Allergy Asthma Clin Immunol.

[9] Bensch GW (2016). Safety of intranasal corticosteroids. Ann Allergy Asthma Immunol.

[10] Corren J (1999). Intranasal corticosteroids for allergic rhinitis: how do different agents compare?. J Allergy Clin Immunol.

[11] Anderson DR (2010). IOP: The Importance of Intraocular Pressure. Pearls of glaucoma management.

[12] Machiele R, Motlagh M, Patel BC (2019). Intraocular Pressure. https://www.ncbi.nlm.nih.gov/books/NBK532237/.

[13] Şimşek A, Bayraktar C, Doğan S, Karataş M, Sarıkaya Y (2016). The effect of long-term use of intranasal steroids on intraocular pressure. Clin Ophthalmol.

[14] Stevens S, Gilbert C, Astbury N (2007). How to measure intraocular pressure: applanation tonometry. Community Eye Health.

[15] Bang SP, Lee CE, Kim YC (2017). Comparison of intraocular pressure as measured by three different non-contact tonometers and Goldmann applanation tonometer for non-glaucomatous subjects. BMC Ophthalmol.

[16] Dereci S, Pirgon O, Akcam M, Turkyilmaz K, Dundar B (2015). Effect of inhaled fluticasone propionate on retinal nerve fiber layer thickness in asthmatic children. Eur J Ophthalmol.

[17] Valenzuela CV, Liu JC, Vila PM, Simon L, Doering M, Lieu JEC (2019). Intranasal corticosteroids do not lead to ocular changes: a systematic review and meta-analysis. Laryngoscope.

[18] Yenigun A, Elbay A, Dogan R, Ozturan O, Ozdemir MH (2018). A pilot study investigating the impact of topical nasal steroid spray in allergic rhinitis patients with dry eye. Int Arch Allergy Immunol.

[19] Mohd Zain A, Md Noh UK, Hussein S, Che Hamzah J, Mohd Khialdin S, Md Din N (2019). The relationship between long-term use of intranasal corticosteroid and intraocular pressure. J Glaucoma.

